# Supplementary material for: The [4Fe-4S] cluster of sulfurtransferase TtuA desulfurizes TtuB during tRNA modification in Thermus thermophilus
Source: Commun Biol. 2020 Apr 7;3:168. doi: 10.1038/s42003-020-0895-3 (PMC7138817; doi:10.1038/s42003-020-0895-3)
Supplement: Supplementary file 1 — Supplementary Information [file 42003_2020_895_MOESM1_ESM.pdf]

Supplementary Figure 1

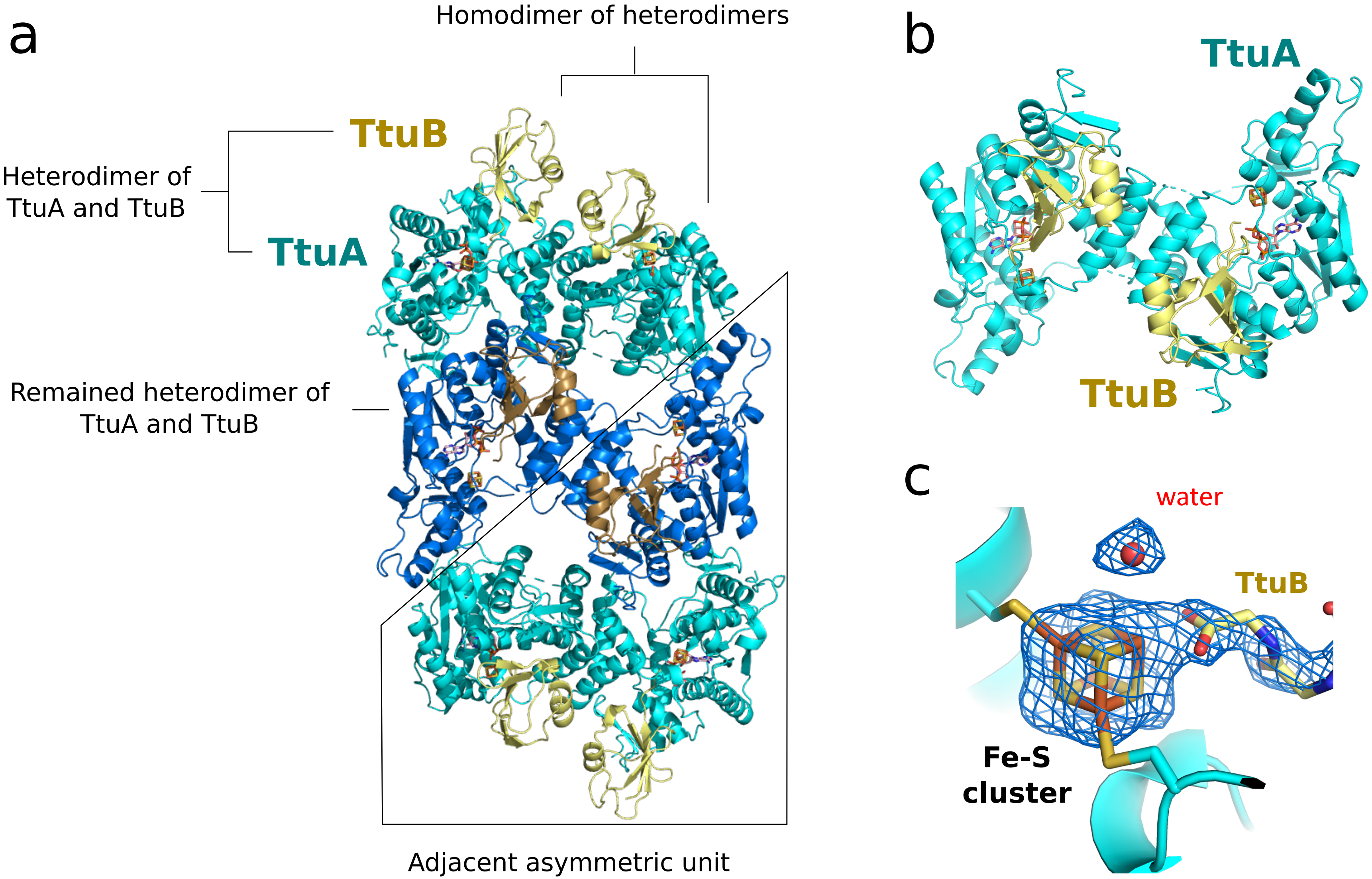

Supplementary Figure 1. Crystal structure of holo-TtuA-TtuB-ATP complex.
(a) The TtuA and TtuB molecules that form a complete (TtuA-TtuB)$_2$ tetramer in one asymmetric unit are colored in cyan and light yellow, respectively. The TtuA and TtuB molecules that form a (TtuA-TtuB)$_2$ tetramer with adjacent asymmetric unit are colored in blue and dark yellow, respectively. The adjacent asymmetric unit is framed by a black trapezoid. (b) Front view of the holo-TtuA-TtuB-ATP complex. (c) Close-up view of the Fe-S cluster in TtuA. The Fe-S cluster is shown as a stick model and colored in orange and yellow. The *Fo*-*Fc* map, which was calculated by omitting the Fe-S cluster and C-terminus of TtuB, is shown as a green mesh (σ=4.0).

Supplementary Figure 2

TtuA -TtuB(COSH) mixture

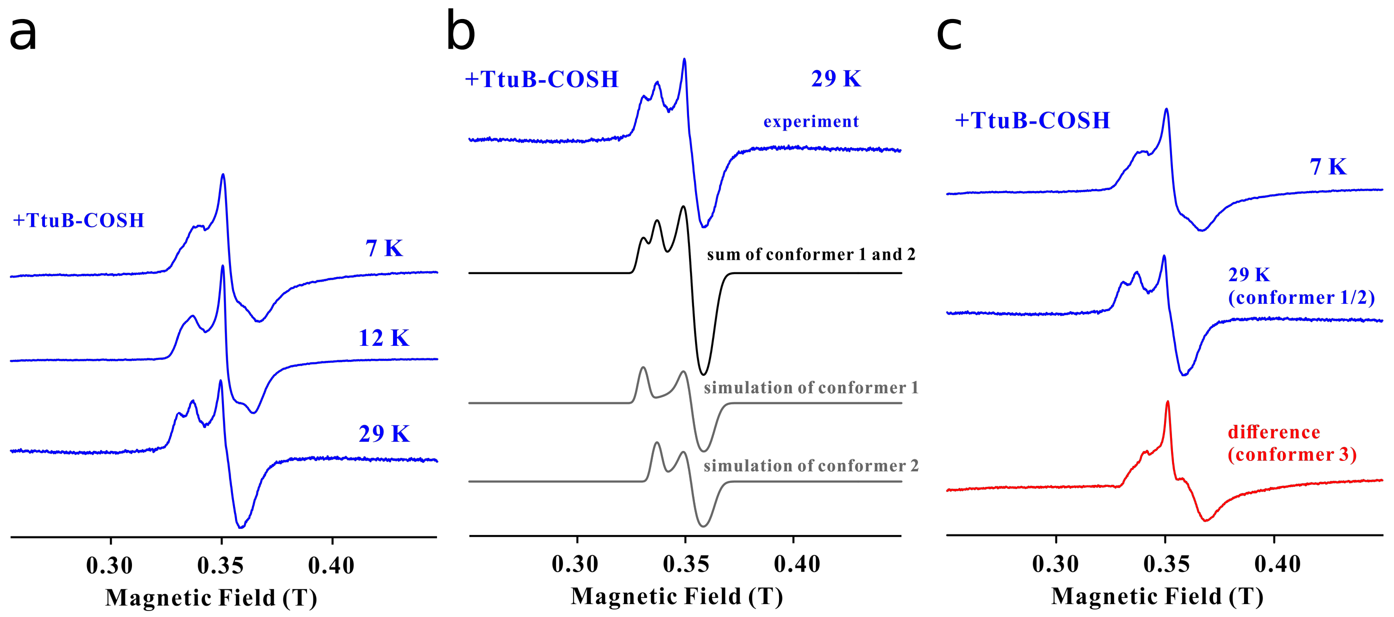

TtuA

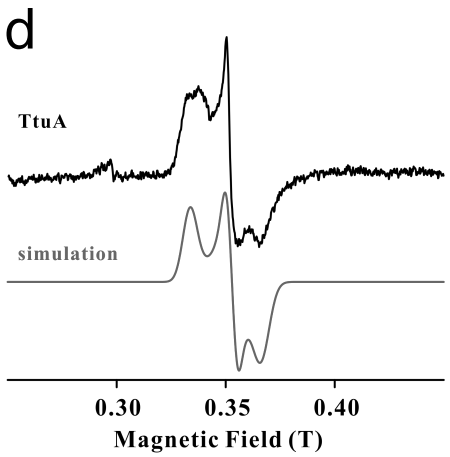

Co-expressed TtuA-TtuB(COOH)

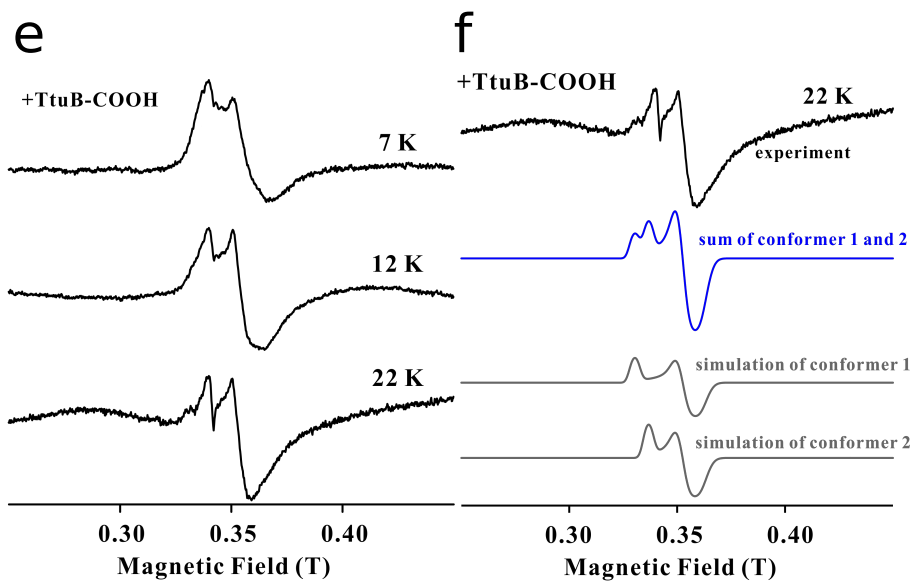

Supplementary Figure 2. EPR spectral analysis of TtuA in complex with TtuB. (a) Temperature dependence of EPR spectra of TtuA in the presence of TtuB(COSH) at different temperatures (7, 12, 29 K). Experimental conditions of EPR are the same as those of Fig. 3a. (b) Deconvolution EPR simulation analysis of the spectrum of TtuA-TtuB(COSH) at 29 K. Simulated signals of conformers 1 and 2 are shown in gray lines (sim 1 and 2), given g = [2.074, 1.943, 1.900] and [2.035, 1.943, 1.900]. Sum 1+2 represents summation of sim 1 and sim 2, and is comparable with the spectrum at 29 K. (c) EPR spectrum for conformer 3 was obtained by subtracting the spectrum at 29 K from that at 7 K. (d) EPR spectrum of TtuA in the absence of TtuB(COSH), in comparison with the simulated signal. (e) Temperature dependence of EPR spectra of the co-expressed TtuA-TtuB(COOH) complex used for crystallization. The spectra observed at 7K, 12 K, and 22 K are shown. (f) Deconvolution EPR simulation analysis of the spectrum of co-expressed TtuA-TtuB(COOH) complex. The simulated signal of conformers 1 and 2 and their summation are shown in the same manner as (b).

Supplementary Figure 3

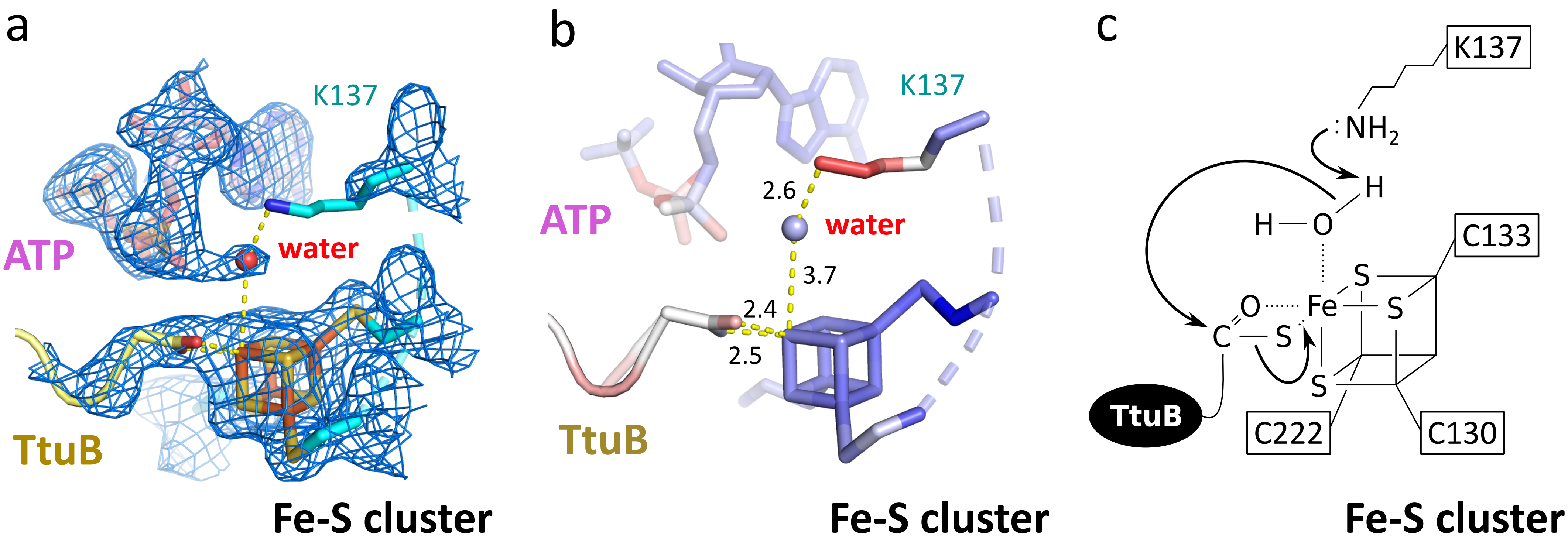

Supplementary Figure 3. Close-up view of the catalytic site.
(a) Crystal structure of the catalytic site of TtuA. The 2*F*o-*F*c map is shown as a blue mesh (σ=2.0).
(b) The catalytic site model is colored according to B-factor (red, 70; blue, 20).
(c) Proposed mechanism of TtuB desulfurization by residue Lys137.

Supplementary Figure 4

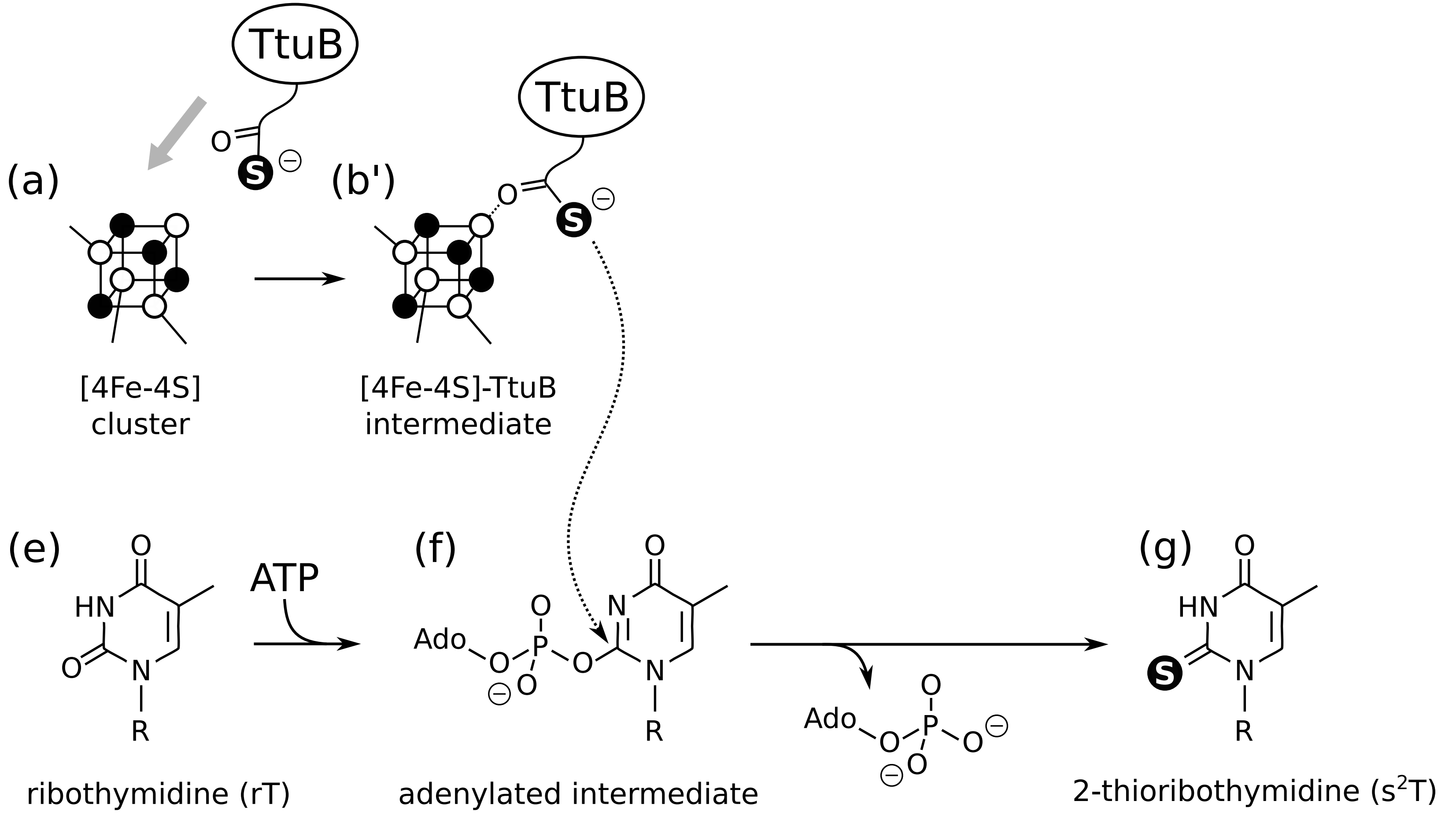

Supplementary Figure 4. Alternative (direct) mechanism of s^2T biosynthesis mechanism.
For easier comparison, all the steps are named in the same manner as Fig.5. Notice that the step (c) and (d) does not exist in the alternative mechanism. And in step (b'), TtuB binds with Fe-S cluster as a monodentate ligand but not bidentate ligand as step (b) in the indirect mechanism. The dotted and curved arrow shows the nucleophilic attack of sulfur to substrate tRNA.

Supplementary Figure 5

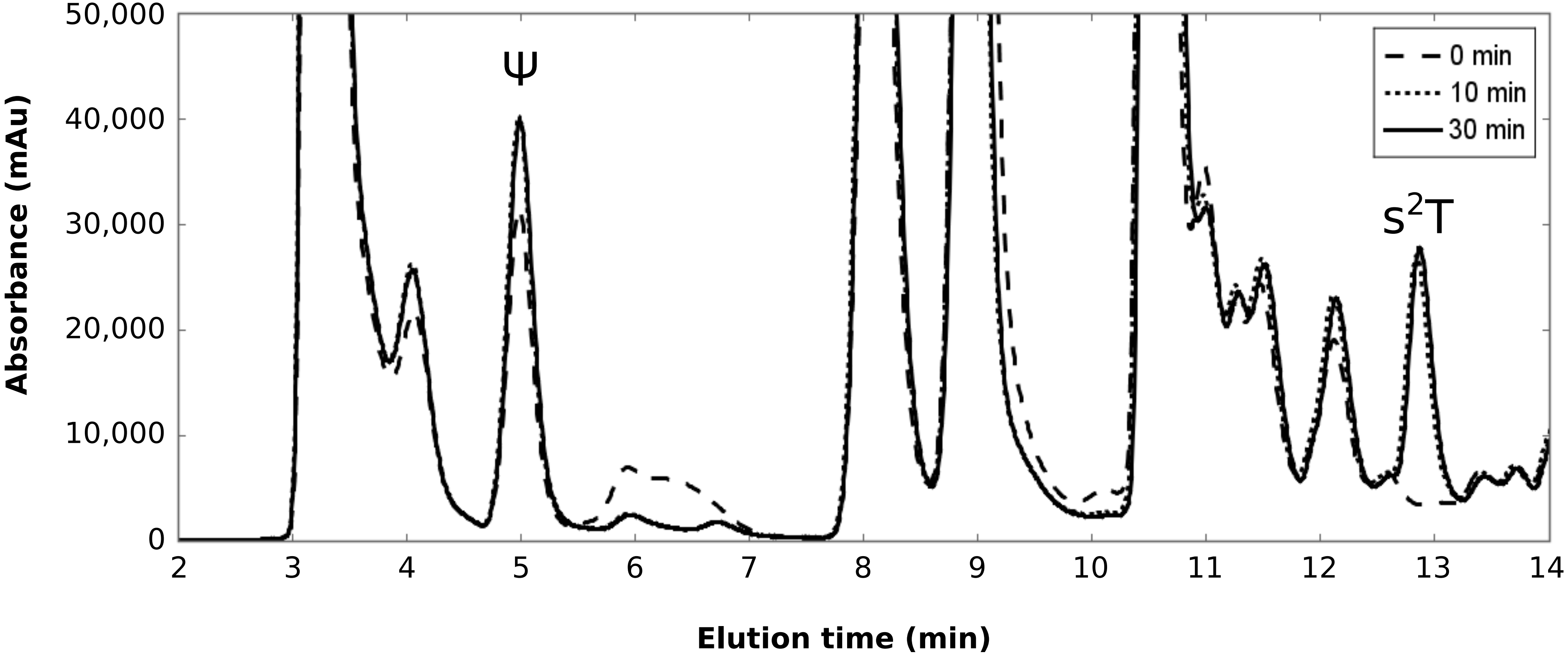

Supplementary Figure 5. Nucleoside HPLC analysis of the modified nucleosides of the reacted tRNA. HPLC chromatograms of the Nuclease P1-treated reaction mixture after incubation for 0, 10, or 30 min are shown by a dashed, dotted, or solid line, respectively. The positions of pseudouridine (Ψ) and 2-thioribothymidine (s^2T) are indicated.

Supplementary Figure 6

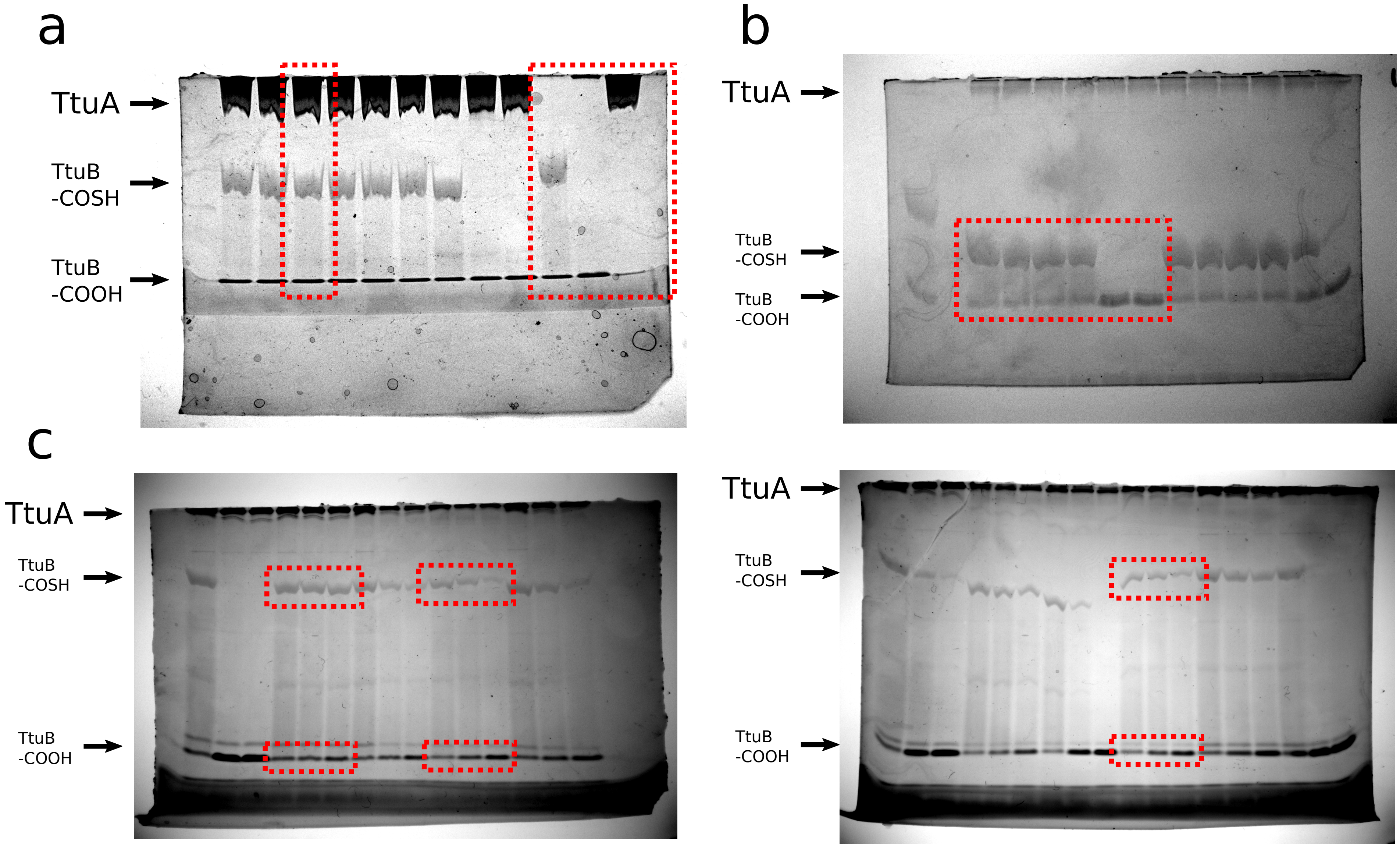

Supplementary Figure 6. Full images of APM-gel used in this study.

(a) The full image of APM-gel shown in Fig. 2. The areas are indicated by red dotted frame. The right area was horizontally inverted in Fig. 2 for convenience of explanation. (b) The full image of APM-gel shown in Fig. 3. The area is indicated by red dotted frame. (c) The full image of APM-gel shown in Fig. 4. The areas are indicated by red dotted frame.

Left: TtuA(S55A), middle: TtuA(D59A), right: TtuA(D161A)
