# Supplementary material for: The [4Fe-4S] cluster of sulfurtransferase TtuA desulfurizes TtuB during tRNA modification in Thermus thermophilus
Source: Commun Biol. 2020 Apr 7;3:168. doi: 10.1038/s42003-020-0895-3 (PMC7138817; doi:10.1038/s42003-020-0895-3)
Supplement: Supplementary file 2 — Descriptions of additional supplementary files [file 42003_2020_895_MOESM2_ESM.pdf]

Supplementary data 1

This file contains raw data of HPLC chromatograms for evaluating s2T formation by TtuA mutants in Fig 5b.

Supplementary data 2

This file contains raw data of HPLC chromatograms for evaluating s2T formation by TtuA mutants in Fig 5c.
